# Human tissue-engineered skeletal muscle: a novel 3D *in vitro* model for drug disposition and toxicity after intramuscular injection

**DOI:** 10.1038/s41598-018-30123-3

**Published:** 2018-08-15

**Authors:** D. Gholobova, M. Gerard, L. Decroix, L. Desender, N. Callewaert, P. Annaert, L. Thorrez

**Affiliations:** 1 Tissue Engineering Lab, Department of Development and Regeneration, KU Leuven, E. Sabbelaan 53, 8500 Kortrijk, Belgium; 2 AZ Groeninge, President Kennedylaan 4, 8500 Kortrijk, Belgium; 3 Drug Delivery and Disposition, Department of Pharmaceutical and Pharmacological Sciences, KU Leuven, O&N II Herestraat 49 - box 921, 3000 Leuven, Belgium; 4 Present Address: Faculty of Physical Education and Physiotherapy, Department of Human Physiology and Sportsmedicine, Building L, Pleinlaan 2, Brussels, Belgium

## Abstract

The development of laboratory-grown tissues, referred to as organoids, bio-artificial tissue or tissue-engineered constructs, is clearly expanding. We describe for the first time how engineered human muscles can be applied as a pre- or non-clinical model for intramuscular drug injection to further decrease and complement the use of *in vivo* animal studies. The human bio-artificial muscle (BAM) is formed in a seven day tissue engineering procedure during which human myoblasts fuse and differentiate to aligned myofibers in an extracellular matrix. The dimensions of the BAM constructs allow for injection and follow-up during several days after injection. A stereotactic setup allows controllable injection at multiple sites in the BAM. We injected several compounds; a dye, a hydrolysable compound, a reducible substrate and a wasp venom toxin. Afterwards, direct reflux, release and metabolism were assessed in the BAM constructs in comparison to 2D cell culture and isolated human muscle strips. Spectrophotometry and luminescence allowed to measure the release of the injected compounds and their metabolites over time. A release profile over 40 hours was observed in the BAM model in contrast to 2D cell culture, showing the capacity of the BAM model to function as a drug depot. We also determined compound toxicity on the BAMs by measuring creatine kinase release in the medium, which increased with increasing toxic insult. Taken together, we show that the BAM is an injectable human 3D cell culture model that can be used to measure release and metabolism of injected compounds *in vitro*.

## Introduction

When testing drug candidates non-clinically, adequate model systems are crucial for a high success rate in clinical trials. Currently, 2D cell culture models are widespread as one of the model systems for high-throughput screening since they are very accessible. However, they lack the natural cell environment. The latter may include cell–cell and cell–extracellular matrix (ECM) interactions as well as mechanical and/or chemical cues^1–4^. Moreover in 2D cell culture, compounds added to the cell culture medium can easily access the target cells and are exposed homogenously to all cells, in contrast to drug exposure to tissues *in vivo*^5,6^. These limitations are well-known and therefore the next step is drug testing in animal models. This allows evaluation of the route of administration, pharmacokinetics and pharmacodynamics. Major drawbacks are the cost of animal experiments and the fact that animal physiology differs from human physiology. Ninety percent of compounds declared safe and effective in animal models subsequently failed in clinical trials, 29% was attributable to toxicity^7,8^. Moreover, current policy promotes replacement, refinement and reduction of animal experiments, the so-called 3 R principle^9,10^. These issues have led to a growing interest in creating 3D non-clinical human tissue models using tissue engineering. In tissue engineering, cells are used with natural or synthetic scaffolds to create *in vitro* bio-artificial tissue that can replace *in vivo* tissue in various applications^11^. Three-dimensional tissue can also be created by differentiation from stem cells. Tissues derived by orchestrated *in vitro* stem cell differentiation are referred to as organoids. Different tissues such as cartilage, bone, heart and skeletal muscle, skin, liver and nerve are being developed^12–18^. Most of the approaches are still in research phase, but some products are already used in clinical practice. For example skin replacement products are already used in burn wound treatment (reviewed in^19^). Besides the use of human *in vitro* grown tissues in regenerative medicine, they can also be used as models for disease aetiology, study of development or for drug testing purposes^20^. For example, commercially available hepatocyte and skin tissue models were described to more accurately reflect the human *in vivo* physiology and have a more representative drug response than 2D cell culture models^21,22^.

Despite the increased interest, many tissue engineering hurdles remain to be tackled. Most of the *in vitro* tissues have relatively small dimensions (often submillimeter) and when used for drug testing they are incubated in a medium containing the drug. Nowadays 12% of the drug formulations from the Belgian drug data base are administered through injection, of which one third intramuscularly^23^. Other injection routes include subcutaneous, intravenous or intradermal^23^. Intramuscular injections deliver medication deep into the muscles, serving as a depot, allowing the medication to be gradually released in the blood. There are several reasons to prefer intramuscular drug administration over oral, subcutaneous or intravenous administration; physicochemical and pharmacokinetic properties of the drug, onset, intensity and duration of the effect and patient’s compliance. When looking at the onset of effect after administration, IV injections produce the most rapid onset of effect, followed by IM and SC injections^24^. So, although the IM route administration is not the fastest way to deliver a drug, it is preferred for drugs that require a longer efficacy. The injection creates a drug depot in the muscle tissue and the drug is released slowly over a certain period of time^25^. For example, longer term low plasma levels were found for the IM administrated antipsychotic drug haloperidol decanoate in contrast to orally administrated haloperidol in schizophrenia patients, limiting side effects^26,27^.

Due to lack of alternatives, candidate drugs and drug formulations for intramuscular administration are tested using methods developed for oral drug administration. *In vitro* dissolution testing of candidate drugs is used to predict *in vivo* drug release profiles^28–31^. But *in vitro* results obtained from these studies cannot adequately predict *in vivo* behavior of intramuscular drug formulations, due to a lack of good representation of the complex muscle physiology. To obtain pharmacokinetic and toxicity data that can be predictive for human drug responses, we studied micro-injection of compounds into a previously developed human bio-artificial muscle (BAM) model^32–34^. The BAM is a muscle bundle consisting of aligned myofibers, able to contract upon electrical and/or mechanical stimulation that has been developed in the Vandenburgh group^34,35^. This model system could bridge the gap between 2D cell culture and animal models, while immediately using human instead of animal tissue. Injection of drugs in engineered tissues has not been described thus far and could be a valuable non-clinical model to assess compound toxicity, metabolism and release profile.

BAMs are formed by mixing muscle cells, isolated from muscle biopsies, in a natural hydrogel in a silicone mold containing two attachment points. Over a one week period, the cell-gel mix contracts to form multinucleated myofibers well-aligned in the direction of the attachment points. Such a model can be used to study mechanisms underlying muscle development, skeletal muscle innervation and vascularization^33,36^. Furthermore this model can be used as an *in vitro* disease model for muscular disorders and drug screening. By using muscular dystrophic muscle cells in the BAM model, Vandenburgh *et al*. were able to establish an *in vitro* assay that could predict the behavior of potential treatments for muscle weakness in Duchenne muscular dystrophy and other muscle disorders^37^. Another example of the potential of the BAM model for drug screening purposes describes the positive effect of insulin-like growth factor 1 and the negative effect of a statin on the morphology and contractility of the human BAM. These effects are also observed in human muscle *in vivo* and show the predictive behavior of the BAM model for skeletal muscle drug screening^38^. While this BAM model has also been evaluated in *ex vivo* gene therapy studies^39^, this model has never been implemented in the context of IM injections. The use of human cells in this 3D muscle model can improve the predictability of the human response on candidate drugs. This would increase the success rate of the compound in later clinical trials and at the same time reduce the cost of the drug discovery process^5^. In this paper we have evaluated myofiber formation in BAMs with myoblasts only (BAM-M) and BAMs with a mixed muscle cell population (BAM – MM), mainly consisting of myoblasts and fibroblasts. We show that several parameters such as compound toxicity, retention, release and metabolism of injected test compounds can be measured in this model system. The effect of myofiber composition in the BAM model on the release profile and metabolism was assessed as well. Two compounds were chosen for injection in the BAMs with an in-house micro-injection system based on the ability to measure the amounts of both released parent compounds and their corresponding metabolites. The first compound; 5(6)-carboxy-2′,7′-dichlorofluorescein diacetate (CDFDA) is hydrolyzed by cellular esterases to 5(6)-carboxy-2′,7′-dichlorofluorescein (CDF), which is a fluorescent compound. The second compound, pro-NanoLuc^®^ substrate, can be reduced by cellular reductases to a NanoLuc^®^ substrate which can subsequently be processed by a NanoLuc^®^ enzyme to produce light. The metabolic reactions that are quantified in the tests, namely hydrolysis and reduction, are two reactions that can occur during drug metabolism. We also determined compound toxicity on the BAMs by measuring creatine kinase release in the medium, which clearly increased with increasing toxic insult. In short, we show that the BAM is an injectable human 3D cell culture model which can reflect compound toxicity and release from muscle-like tissue and is capable of metabolizing the injected compounds, a process that is also relevant to the *in vivo* situation.

## Methods

### Cell culture

Human skeletal muscle myoblasts (M) from a skeletal muscle biopsy from the abdomen of a 28-year old diabetic Caucasian man were acquired from Tebu-Bio, 088SKB-F (lot SKM070512A), certified to contain 94.8% CD56-positive cells at passage 3. Cells were expanded from passage 3 to 4 in a T75 cell culture flask (Elscolab) and from passage 4 to 7 in T175 cell culture flasks in Skeletal muscle Growth Medium (SkGM, Lonza), supplemented with 15% Fetal bovine serum (FBS, Fisher Scientific #10500064). Cells were split [4000 cells/cm2] at approximately 70% confluence with 0.125% trypsin/EDTA (Thermo Fisher Scientific #25200056). A human skeletal muscle cell population was isolated from a biopsy from the vastus lateralis muscle of a 60-year old Caucasian man 2 days post-mortem (Human Body Donation programme of KU Leuven University, Prof. E. Vereecke) as described in^40^. In the mixed muscle (MM) cell population, myoblast percentage and fusion index were determined as described in^33^. Cells were expanded until 12 doublings in T175 cell culture flasks in SkGM, supplemented with 15% FBS and split (4000 cells/cm^2^) at approximately 70% confluence with 0.125% trypsin/EDTA. C2C12 mouse myoblast cell line was acquired from Sigma. Cells were expanded in C2C12 growth medium: DMEM Glutamax (Thermo Fisher #31966021), 10% FBS, 1% Penicilin/Streptomycin (P/S, Thermo Fisher #15070063). Cells were split at approximately 90% confluency with 0.125% trypsin/EDTA.

### Human muscle strip isolation

A human muscle tissue biopsy, 307 mg wet weight, from the vastus lateralis muscle of a 60-year old Caucasian woman was isolated 1 day post-mortem (Human Body Donation programme of KU Leuven University, Prof. E. Vereecke). Muscle tissue was transferred to a 50 ml tube with chilled 20 ml DMEM Glutamax + 1% P/S. Working in a biological safety cabinet, muscle tissue was transferred to a 60 mm petri dish with 2 ml DMEM Glutamax + 1% P/S. Using sterile forceps and scalpel, excess connective tissue and fat was removed from the tissue sample. Then, the muscle tissue was cut into approximately 2 mm × 10 mm strips. Using 1.2 cm long sterile stainless steel insect pins (Vermandel #20.051), muscle strips were pinned into sterile sylgard coated 6-well plate wells under tension, with one pin at each end of strip. Strips were cultured for 1 day in DMEM Glutamax + 1% P/S until injection.

### Bioengineering of BAMs

BAMs were engineered as previously described^33,41^. Briefly, C2C12 mouse myoblasts, human myoblasts (M) or human mixed muscle (MM) cells were trypsinized with 0.125% trypsin/EDTA, counted and mixed with 500 µl human thrombin (Bio Pharm Laboratories LLC #SKU 93–050, 4 U/ml in DPBS (+Ca^2+^, +Mg^2+^) (Gibco #14040-083)). The mixture was cast into 25-mm-long silicone rubber molds with end attachment sites spaced 20 mm. Then, 500 µl fibrinogen (Merck Chemicals #341576, 2 mg/ml in DPBS (with Ca^2+^ and Mg^2+^)) was added and the cell-gel mix was mixed by quickly pipetting up and down, to form a fibrin hydrogel (1 mg/ml) containing the M or MM cells. Following two hours incubation at 37 °C, SkGM medium for human M and MM cells and C2C12 growth medium for C2C12 cells supplemented with fibrinolysis inhibitors aprotinin (92.5 µg/ml in PBS (Gibco #10010-015, Carl Roth #A162.3) and tranexamic acid (400 µM in PBS, Sigma #857653-10 G) was added. Two days after casting, the medium was switched to differentiation medium, SkFM, in the case of human M and MM cells: DMEM GlutaMax with 10 ng/ml hEGF (Peprotech #AF-100-15), 10 µg/ml insulin (Sigma-Aldrich #I9278), 50 µg/ml BSA (Sigma-Aldrich #A2153) and 50 µg/ml gentamicin (Thermo Fisher #15750037), also containing fibrinolysis inhibitors. For C2C12 cells the C2C12 growth medium was switched to C2C12 fusion medium containing DMEM Glutamax, 2% Horse serum, 1% P/S, fibrinolysis inhibitors and Cytosine β-D-arabinofuranoside (Sigma #C1768). Medium was replaced every 2 days and BAMs were kept in culture for 7 days prior to microinjection.

### Injected compounds

5(6)-carboxy-2′7′-dichlorofluorescein diacetate (CDFDA, Sigma Aldrich #21884) was freshly dissolved in DMSO (Sigma Aldrich, #D2650) to 100 mM and further diluted in 30% polyethylene glycol-400 (Sigma Aldrich #202398) in HBSS without Ca^2+^ and Mg^2^ (Thermo Fisher # 14175053) to a final concentration of 500 µM for microinjection. 5(6)-carboxy-2′7′-dichlorofluorescein (CDF, Sigma Aldrich # 21882) was freshly dissolved in DMSO (100 mM) and diluted to a final concentration of 250 µM in HBSS. The pro-NanoLuc® substrate of the RealTime-Glo™ MT Cell Viability Assay (Promega #G9711) was used as a luminescent micro-injectable substrate. Mastoparan (Sigma Aldrich #M-5280), a wasp venom peptide, was dissolved to 1 mg/ml in PBS.

### Microinjection

**Figure 1:**
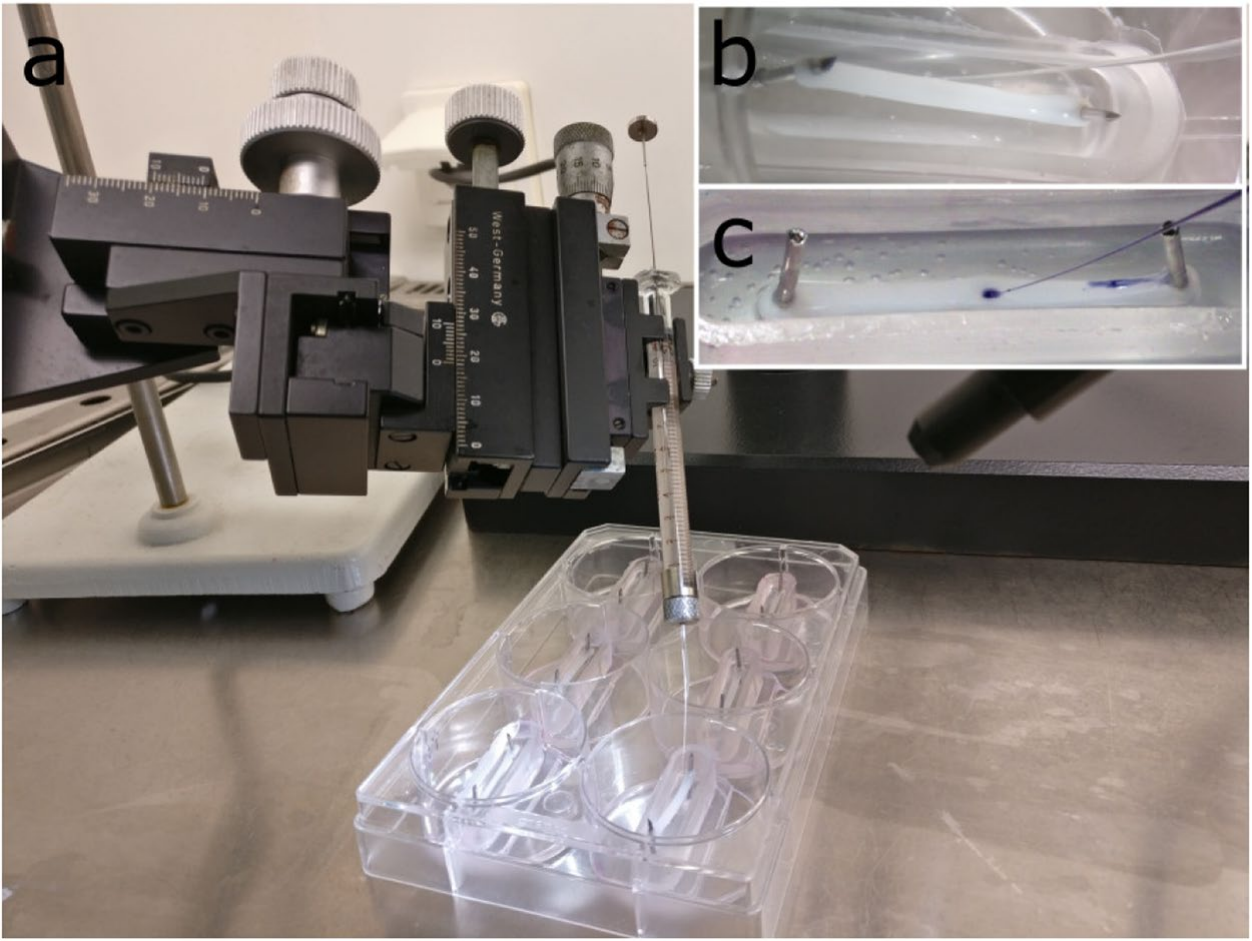
Microinjection setup. (**a**) In-house micro-injection setup. A pulled glass needle is attached to a 10 µl Hamilton syringe which can be accurately positioned in X-, Y- and Z-direction with a stereotactic device. Inlay images (**b** and **c**) show the enlarged view as seen through the stereomicroscope of the injection of pro-NanoLuc® substrate (**b**) or two trypan blue injections (**c**) in BAMs.

Two hours before injection, BAMs or human muscle strips were washed twice with 1 ml HBSS and further incubated in HBSS for two hours. Immediately before injection, BAMs or strips were washed again with 1 ml HBSS. Next, HBSS was removed to perform the injection. An in-house microinjection system was built using a syringe (Hamilton® Syringe 75RN 5.0 µL) fitted with a glass capillary using a 1 mm compression fitting (Dual Ferrule RN adaptor, Hamilton). The 1 mm glass capillary (VWR #612-2804) was pulled by hand and cut at the end to have an external diameter of 10–30 µm. The syringe with glass capillary could be exactly positioned using a 3D stereotactic system. Visual monitoring of penetration of the capillary in the BAM was performed with a stereomicroscope (Wild MSB, Heerbrugg Switzerland) (Fig. 1a). Each BAM or strip was injected 1 to 4 times with 0.5–1 µl per sample (Fig. 1b,c). For visualization and optimization of the injection procedure, Trypan blue (Thermo Fisher) was used. In case of multiple injections, different areas of the BAM were injected. Immediately after injection, 800 µl HBSS was added to the mold, completely submerging the BAM or muscle strip in HBSS. The tissue was washed twice with 800 µl HBSS, each time with a fixed time interval between washes (respectively 1 and 1.5 min) after which again 800 µl HBSS was added. All compounds in the washing steps were considered to be direct reflux. Effectively injected compound was calculated by subtracting the amount of refluxed compound from the total amount of compound that was recovered. At 5 min, 1.5 h, 16 h and 40 h 800 µl samples were taken and 800 µl fresh HBSS was added. The HBSS samples were stored for further processing at 4 °C for pro-NanoLuc® substrate injections and at 4 °C in acidic conditions for CDFDA injections (addition of 15 mM HCl per sample) or −20 °C for HBSS and mastoparan injections. Measurements of CDF and NanoLuc® substrate occurred within 48 hours after collection of the final samples.

After the experiment, the BAM diameter was measured with a micrometer, each BAM was removed from its attachment sites, dried for 1 min on a tissue and frozen at −20 °C. To determine whether residual compound could be found in the BAMs, they were disrupted using a tip sonicator (Branson, 5–10 cycles of 12 micron, 10 s each) in 800 µl RIPA buffer (25 mM Tris pH 7.4, 150 mM NaCl, 1% Nonidet^TM^ P40 (Igepal)) while kept on ice. This 800 µl sample was further processed as the HBSS samples mentioned above.

### CDFDA incubation in 2D cell culture

50,000 M or MM cells were cultured in SkGM in 6-wells to 80% confluence and then switched to SkFM medium to induce myofiber formation. Cells were washed twice with 1 ml HBSS. 800 µl of 625 nM CDFDA solution in HBSS was added to cells. At 5 min, 1.5 h, 16 h and 40 h, 800 µl samples were taken and the same volume of HBSS was added.

### Creatine kinase measurement

The toxicity of an injected compound was determined by measuring creatine kinase (CK) release. For toxicity measurements, the 3 types of BAM (mouse C2C12, human BAM-M and human BAM-MM) were injected as mentioned above with mastoparan at a myotoxic concentration of 1 mg/ml^42^ or with HBSS. After injection, 800 µl of HBSS was added to the silicone mold of the BAM. The plates containing the BAMs were incubated at room temperature for 5 minutes after which the HBSS, containing the CK that was released by the BAMs, was stored at −20 °C until CK measurement. As a positive control, BAMs were crushed in a cell homogenizer (Kimble Kontes) in 800 µl HBSS. CK concentration was measured on a Roche Cobas c501 chemistry analyzer with the CKL test kit (Roche Diagnostics #04524977) by measuring NADPH absorbance proportional to CK concentration. The lower quantification limit of this assay is 7 U/l.

### Detection of CDF fluorescence

A calibration curve was prepared by serial 2x dilution of CDF and CDFDA in HBSS, starting at 250 µM. 150 µl of all samples was added in duplicate in a white 96-well plate (Roche #004729692001). The plate was shaken for 30 seconds and CDF fluorescence was measured using an excitation wavelength of 490 nm, emission was captured between 510 and 570 nm (GloMax®-Multi Microplate Multimode Reader, Promega) three times for each sample. For CDFDA determination, in half of the samples, 6 µl of NaOH (2 M) was added to increase the pH above 11 and the plate was shaken for 30 seconds. This induces rapid and complete conversion of all CDFDA to CDF (data not shown). After 30 min incubation at room temperature, CDF fluorescence was recorded again as specified above. The amount of CDFDA was calculated as the total amount of CDF after forced hydrolysis minus the amount of CDF before hydrolysis.

### Detection of NanoLuc^®^ substrate luminescence

The pro-NanoLuc® substrate (Promega, #N1110) is supplied as a 1000x concentrated solution, to be diluted to a 1x solution for use in cell culture as advised by the manufacturer. Absolute concentration is proprietary so all concentrations are indicated as an x-times concentrated solution. As a calibration curve, a 2x dilution series was made of the pro-NanoLuc® substrate in HBSS, starting with a 0.5x concentration. 100 µl of each sample was pipetted in the white 96-well plate. All samples were added in duplicate and a 0.25x concentration of the NanoLuc® enzyme was added to each well. In one half of the samples and in the wells of the calibration curve, 20 mM dithiothreitol (DTT) (AppliChem #A2948) was added to induce forced reduction of the pro-NanoLuc® substrate in the sample. The plate was shaken for 30 s and then incubated at 37 °C for 2 h. Luminescence occurs by conversion of the reduced Nanoluc® substrate by the NanoLuc® enzyme and is proportional to the amount of reduced substrate. Luminescence was recorded with an integration time of 1 s on a GloMax®-Multi Microplate Multimode Reader (Promega).

### Immunocytochemistry

Immunocytochemistry was performed in order to characterize the myoblast (M) and mixed muscle cell (MM) population used in the injected BAMs. The percentage of myoblasts and their fusion index was determined by a desmin and tropomyosin immunofluorescence staining. For desmin staining, myoblasts were cultured in SkGM in 24-well dishes for 2–3 days until ∼70% confluent. Fixation was performed in a 1:1 methanol-aceton mix at −20 °C for 10 min. For determination of fusion index, the cells were cultured to 80% confluence in SkGM and then switched to SkFM for 4 days to induce fusion into myofibers. Fixation was performed in 4% formaldehyde freshly prepared from paraformaldehyde (Merck # 1040031000) at RT for 10 min. Next, cells were permeabilized in blocking buffer containing 0.2% Triton-X-100 (Sigma #X100) and 1% bovine serum albumin (BSA, Sigma # A2153) in PBS (30 min, RT). Subsequently, cells were incubated overnight at 4 °C with a monoclonal mouse antibody against desmin (Sigma, D1033, 1:200 in blocking buffer) or tropomyosin (Sigma, T9283, 1:100 in blocking buffer) to determine the myoblast percentage and the fusion index respectively. Cells were labeled with a polyclonal goat anti-mouse secondary antibody (Alexa Fluor 568, Invitrogen # A11004, 1:200 in PBS) for 30 minutes in the dark and subsequently incubated with DAPI (0.1 µg/ml in PBS, Life Technologies) for 1 hour. Images were acquired with Zeiss Zen software by AxioCam ICc 1 camera mounted on a Zeiss Axiovert 10 microscope. The percentage of myoblasts in a muscle cell population was defined as the ratio of desmin positive cells to the total number of cells (identified by the DAPI-stained nuclei). For each replicate 150–300 cells were counted. Fusion index was defined as the ratio of nuclei in tropomyosin positive cells to the total number of myoblast nuclei in the population.

### Whole mount immunofluorescence staining

The 3 types of BAMs (mouse GFP-labeled C2C12, human BAM-M and human BAM-MM) and the human muscle strips were washed (3 × 5 min in PBS) and then removed from the attachment sites. Then, they were pinned on Styrofoam to preserve their original shape during fixation in 4% formaldehyde for 1 h and stored at 4 °C in PBS. Immediately before whole-mount staining, samples were fixed a second time in −20 °C methanol for 10 minutes and permeabilized in blocking buffer for 3 hours. Subsequently, the human BAMs and muscle strip were incubated overnight at 4 °C with a monoclonal mouse antibody against tropomyosin (Sigma, T9283, 1:100 in blocking buffer) and MF20 (DSHB), recognizing all myosin heavy chain (MHC) isoforms. Next, the human BAMs and muscle strips were washed and incubated with a polyclonal goat anti-mouse secondary antibody (Alexa Fluor 633, A-11059 or A-21063, Invitrogen, 1:200) for 30 minutes in the dark, followed by incubation with DAPI (Life Technologies, 0.1 µg/ml in PBS) for 1 hour. Samples were stored in PBS in the dark until visualized.

### Confocal imaging and data analysis

Fixed GFP-labeled C2C12 BAMs, fixed and stained human BAMs or muscle strips were placed on coverslips and visualized by confocal microscopy (Zeiss LSM710) within 48 hours. Per sample, 2–5 z-stacks were acquired, each containing 20–40 images (depending on the intensity of the signal at various depths) every 5 µm towards the center of the tissue. For myofiber analysis in C2C12 BAMs, z-projections of 20 µm were created in ImageJ^43^. The 20 µm z-projections were analyzed for different parameters of myofiber formation: myofiber alignment, length and myofiber density per microscopic field. Myofiber alignment was determined by the standard deviation of the angles of the myofibers compared to the mean alignment direction. A lower number thus indicates better alignment. In human BAMs number of nuclei per fiber were counted on 2D images.

### Quantitative real-time PCR

RT-PCR was carried out to determine the expression of the different myosin heavy chain isoforms (*MYH1, MYH3, MYH8*) and myogenin (*MyoG*) in the 2 types of human BAM and muscle strip to compare the developmental state of the myofibers in the tissue-engineered constructs to mature human muscle. To normalize for the amount of cells, 3 reference genes were used: *GAPDH, HSP90AB1* and *RPL13A*. Therefore BAMs or muscle strips were washed twice with PBS for 5 min and removed from the attachment sites. Per sample, 1 ml lysis buffer (Invitrogen #46-6001) with 1% 2-mercaptoethanol (Janssen Chimica #1254734) was added for 5 minutes at room temperature. The samples were stored at −80 °C until further processing. The samples were disrupted using a tip sonicator (Branson, 5–10 cycles of 12 micron, 10 s each) while kept on ice and then centrifuged for 5 min at 2600 g. Total RNA was isolated from supernatans using the PureLink RNA Mini Kit (Ambion #12183018 A). 2 µg of isolated RNA was reverse transcribed with the qScript cDNA SuperMix (Quantabio #95048-100) in 20 µl. PCR was performed with GeneAmp® PCR System 9700 (Applied Biosystems). Gene-specific primers (Table 1) were designed using NCBI/Primer-Blast and Primer3plus. Primer efficiency was determined by running a qRT-PCR for each specific gene primer pair with a serial dilution of cDNA. QRT PCR was performed with the LightCycler® 480 Instrument (Roche) using Perfecta Sybr Green Supermix (Quantabio #95054-500). All samples were run in duplicate. 40 cycles were run with a DNA dissociation step (95 °C, 15 sec”), a primer annealing step and an amplification step (60 °C, 45 sec”). After each cycle, Sybr Green fluorescence was recorded. After 40 cycles, a melting curve was recorded in which the temperature was increased at 1 °C per sec” from 60 °C to 95 °C while the Sybr Green fluorescence was recorded continuously.

**Table 1:** List of primers used for qRT-PCR.

Gene	Orientation	Primer sequence (5′−3′)	Amplicon size (bp)
*MYH1*	Forward	GGG AGA CCT AAA ATT GGC TCA A	106
	Reverse	TTG CAG ACC GCT CAT TTC AAA	
*MYH3*	Forward	CTT GTG GGC GGA GGT CTG G	119
	Reverse	AGC TAT GCC GAA CAC TTC CAT	
*MYH8*	Forward	ACA TTA CTG GCT GGC TGG AC	143
	Reverse	ACC TTT CTT CGC GCT GCT AT	
*MyoG*	Forward	GTG TGT AAG AGG AAG TCG GTG TC	90
	Reverse	GAA GGC CTC ATT CAC CTT CTT	
*GAPDH*	Forward	TCA AGA AGG TGG TGA AGC AGG	168
	Reverse	ACC AGG AAA TGA GCT TGA CAA A	
*HSP90AB1*	Forward	AGA AAT TGC CCA ACT CAT GTC C	75
	Reverse	ATC AAC TCC CGA AGG AAA ATC TC	
*RPL13A*	Forward	CCT GGA GGA GAA GAG GAA AGA GA	126
	Reverse	TTG AGG ACC TCT GTG TAT TTG TCA A	

### Statistics

Number of replicates refers to number of analyzed images for microscopic analyses, to the number of wells containing 2D cells treated with a compound, to the number of biological samples for quantitative real-time PCR analysis or to the number of BAMs for thickness analyses and microinjections. D’Agostino & Pearson normality test and Bartlett’s test were used to verify normality of the data and equality of variances, respectively. Normally distributed data with equal variances were analyzed by an unpaired Student’s t-test when two groups were compared. For comparing several normally distributed groups, a one-way ANOVA was used with a Bonferroni multiple comparison post test. When taking into account the factor time and cell type, a two-way ANOVA was used when comparing several groups. For groups that were not normally distributed and/or had unequal variances, a non-parametric Mann-Whitney test was used for comparison between two groups while Kruskal-Wallis test followed by a Dunn’s post test was performed for multiple comparisons. All values were expressed as mean ± standard deviation. Relative mRNA expression was statistically analyzed with relative expression software tool (REST 2009, Qiagen). The REST software calculates whether gene expression is significantly different between the sample and control group using bootstrap randomization^44^. Relative expression data were displayed in Whisker boxplots. *p < 0.05, **p < 0.01, ***p < 0.001.

### Data availability

The datasets generated and analysed during the current study are available from the corresponding author on request.

## Results

### Optimization of the microinjection procedure

BAMs and muscle strips were injected with microliter quantities using a custom-built injection device (Fig. 1a,b). In order to obtain a 3D microinjection system, we characterized and optimized different parameters. First, the thickness of the BAM can influence the injection. The thicker the BAM, the easier the injection can be performed and the higher the maximal injection volume. Increasing the thickness of the BAM is possible by increasing the amount of extracellular matrix protein used when casting the cell-hydrogel mix in the mold. However, increasing the concentration of fibrinogen to 2 mg/ml in C2C12 mouse BAMs, caused a decrease in the quality and the amount of muscle fibers, especially in the center of the BAM (Supplementary Figure S1); therefore, 1 mg/ml fibrin was further used throughout experiments. Using 4.10^6^ C2C12 cells per BAM instead of 2.10^6^ cells did not further improve the myofiber quality (Figure S1). The thickness of BAMs engineered with 2.10^6^ human cells in 1 mg/ml fibrin was 1.78 mm ± 0.18 mm (BAM-M) and 1.45 ± 0.14 mm (BAM-MM). Muscle strips used for toxicity testing and CDFDA injections were 1.41 ± 0.31 mm thick. Due to these thickness restrictions, glass capillary needles with a small outer diameter were preferable for injection to commercial stainless steel needles, which have an outer diameter of >100 µm. Hand-pulled glass capillary needles with different outer diameters were fitted on a 5 µl syringe. Initial experiments to monitor the injection process were performed with Trypan blue dye (Fig. 1c). Using a capillary needle with an outer diameter of 10–30 µm ensured efficient injection without clogging the needle and allowed easy penetration into the elastic tissue-engineered muscle. Using Trypan blue, we determined that the injected volume should be smaller than or equal to 1 µl to avoid leakage of the dye from the BAM during injection. However, to increase the injected amount of compound/dye, we were able to inject each BAM up to 5 times without creating physically overlapping injection sites. In Fig. 1a, the microinjection setup is shown. When injecting with Trypan blue, we could also observe an amount of dye immediately exiting the BAM when removing the needle. Therefore, the BAM was washed twice after each injection to remove this refluxed compound. Finally, when analyzing the release behavior of one of the studied compounds, CDFDA, we also determined that a fraction of the CDFDA adhered to the silicone mold (Supplementary Table S1), indicating an influence of the used materials on the recovered amount.

### Characterization of myoblast (M) and mixed muscle (MM) cell populations

**Figure 2:**
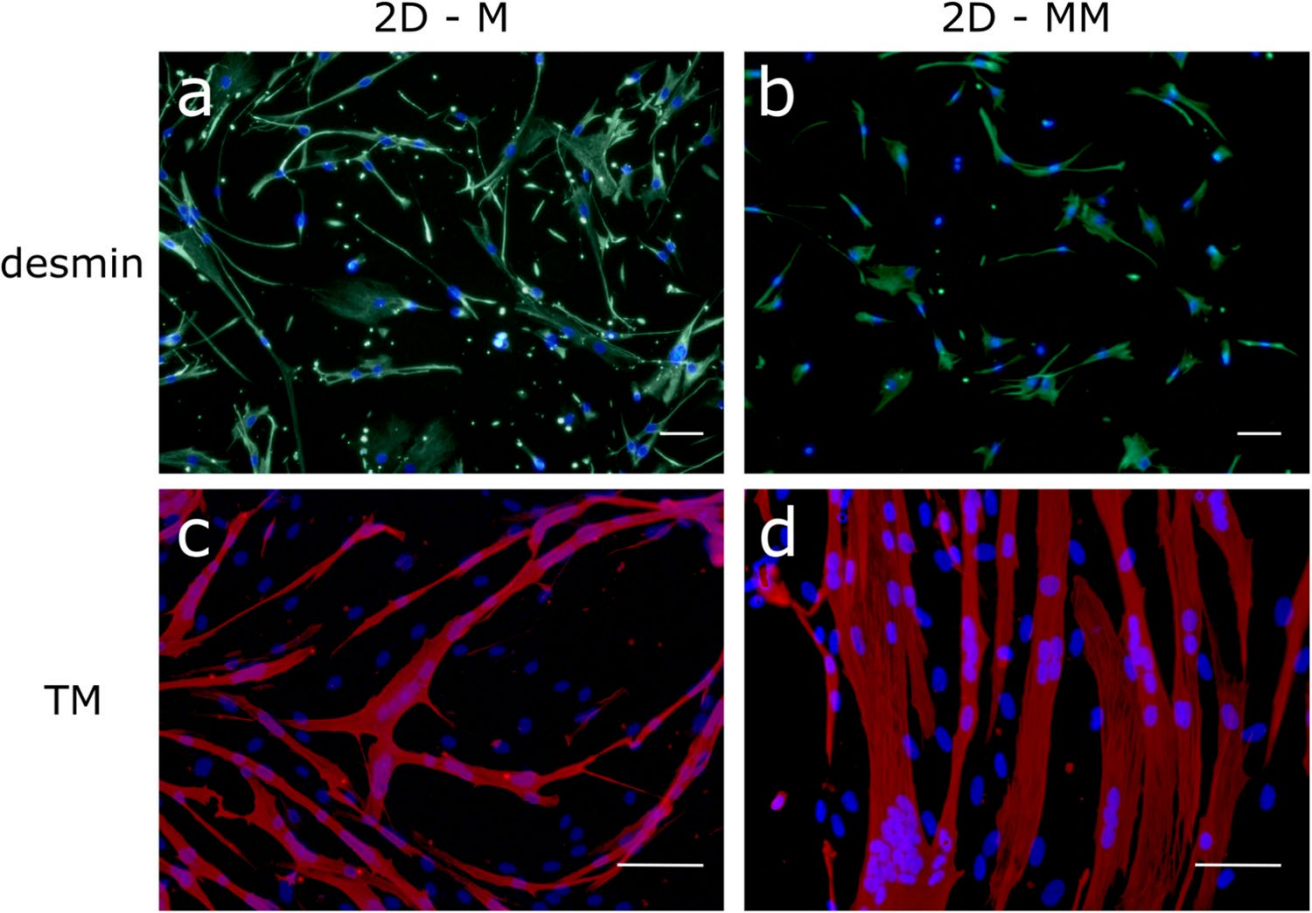
Human cells used for microinjection are highly myogenic and capable of differentiation into muscle fibers. (**a**,**b**) The cell population with only myoblasts (M) and mixed muscle cell population (MM) contains 100% and 82 ± 5% myoblasts respectively as shown by desmin immunofluorescence staining (green) and nuclear staining (DAPI, blue) (a and b respectively). (**c**,**d**) After four days in SkFM, positive tropomyosin staining (red) and cell fusion indicates efficient differentiation to muscle fibers in the M and MM cell populations. Scale bars represent 50 µm.

The cell population with only human skeletal muscle myoblasts (M) was characterized by desmin staining at passage 4, showing that the population contained 98 ± 2% desmin positive cells, consistent with 95% CD56^+^ cells at passage 3 as described in the datasheet, both CD56 and desmin being myogenic markers. The cells were further expanded in growth medium and used for tissue engineering at passage 7. At this passage, cells were 100% desmin positive (Fig. 2a) and had a fusion index of 64 ± 3%, forming muscle fibers with 8 ± 1 nuclei per fiber (n = 5) (Fig. 2c). The mixed muscle (MM) cell population, obtained when the isolated muscle cell population is not further selected, contained 82 ± 5% desmin positive myoblasts (n = 6) (Fig. 2b). Non-myogenic cells, which were previously reported to be mainly fibroblasts^45,46^, contributed to the remaining 18%. The myoblasts in the MM cell population had a fusion index of 80 ± 15%, forming muscle fibers with 10 ± 4 nuclei per fiber (n = 6) (Fig. 2d).

### Myofiber formation in BAMs

**Figure 3:**
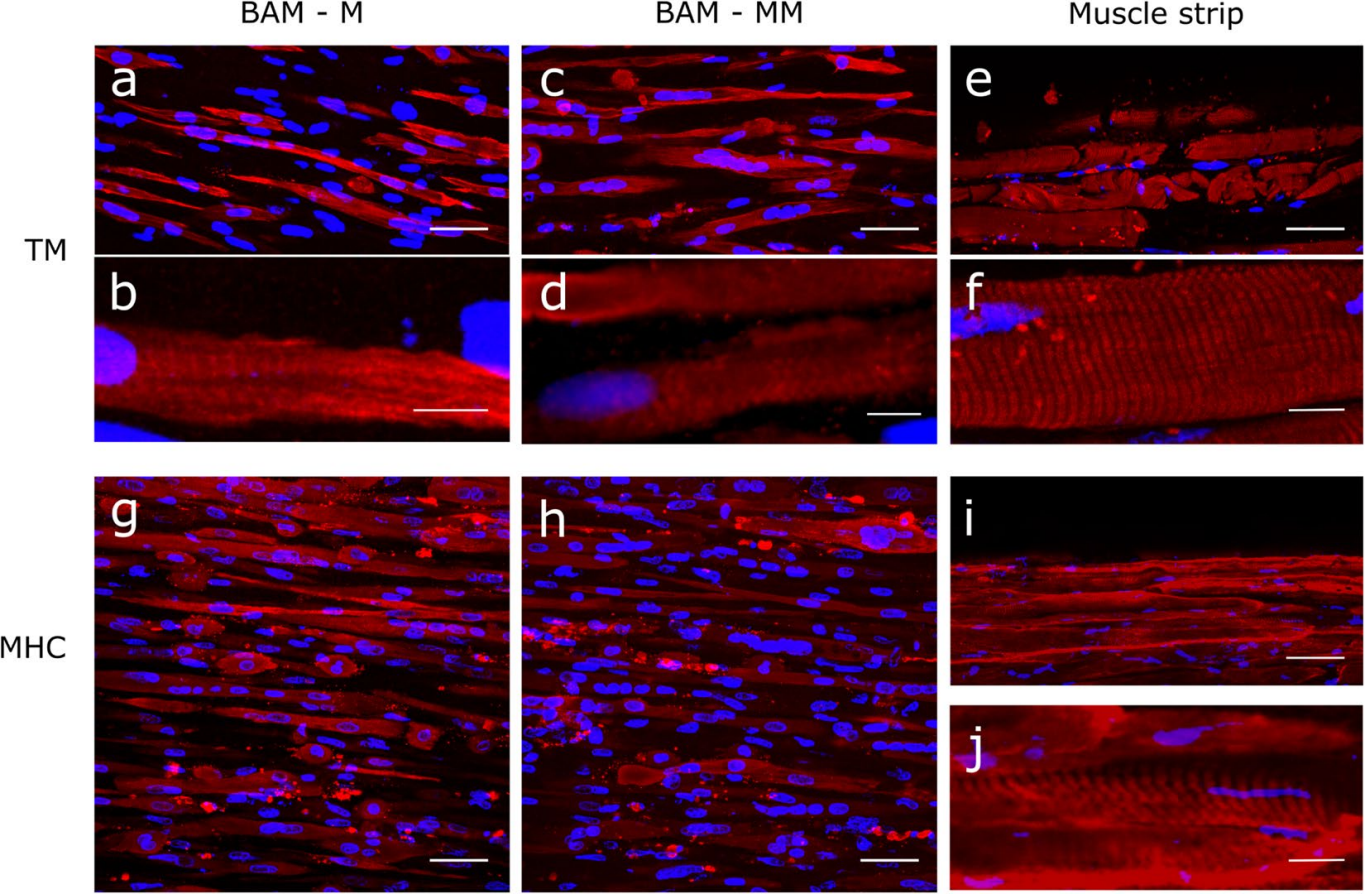
Myofibers within BAM-M and BAM-MM are well-aligned, multinucleated and occasionally striated. (**a**–**f**) Confocal microscopy images of tropomyosin (TM) immunofluorescence stained myofibers in 1 week old 3D BAMs with myoblasts only (BAM-M) (**a**,**b**), in 1 week old BAMs with mixed muscle cell population (BAM-MM) (**c**,**d**) and isolated human muscle strips (**e**,**f**). (**g**–**j**) Confocal image of MHC immunofluorescence stained myofibers in BAM-M (**g**), 1 week old BAM-MM (**h**) and muscle strips (**i**,**j**). The BAMs contain well-aligned, multinucleated (DAPI, blue), occasionally striated myofibers (**b**,**d** and **f**). Scale bars represent 50 µm (**a**,**c**,**e**,**g**,**h** and **i**) and 10 µm (**b**,**d**,**f** and **j**).

BAMs were formed by mixing 2 × 10^6^ cells in a 1 mg/ml fibrin matrix in a silicone mold containing 2 attachment points. During the course of 1 week, the cell-gel mix contracted to form BAMs. Myofiber formation was assessed with tropomyosin (TM) and myosin heavy chain (MHC) immunofluorescence in BAMs with myoblasts only (BAM-M) and in BAMs with a mixed muscle cell population (BAM – MM) in comparison to human muscle strips. In both BAM conditions, TM (Fig. 3a–d) and MHC (Fig. 3g,h) positive muscle fibers were observed, well-aligned in the direction of the attachment points (Fig. 3). Presence of striated myofibers indicates an organized contractile aparatus as shown in human muscle strips (Fig. 3f and j). Striation was also observed in the BAMs although to a lesser extent (Fig. 3b and d). In human muscle strips nuclei were at the periphery (Fig. 3e and i), while in the tissue engineered constructs in the middle of the myotubes (Fig. 3a,c,g,h).

### Muscle gene expression

**Figure 4:**
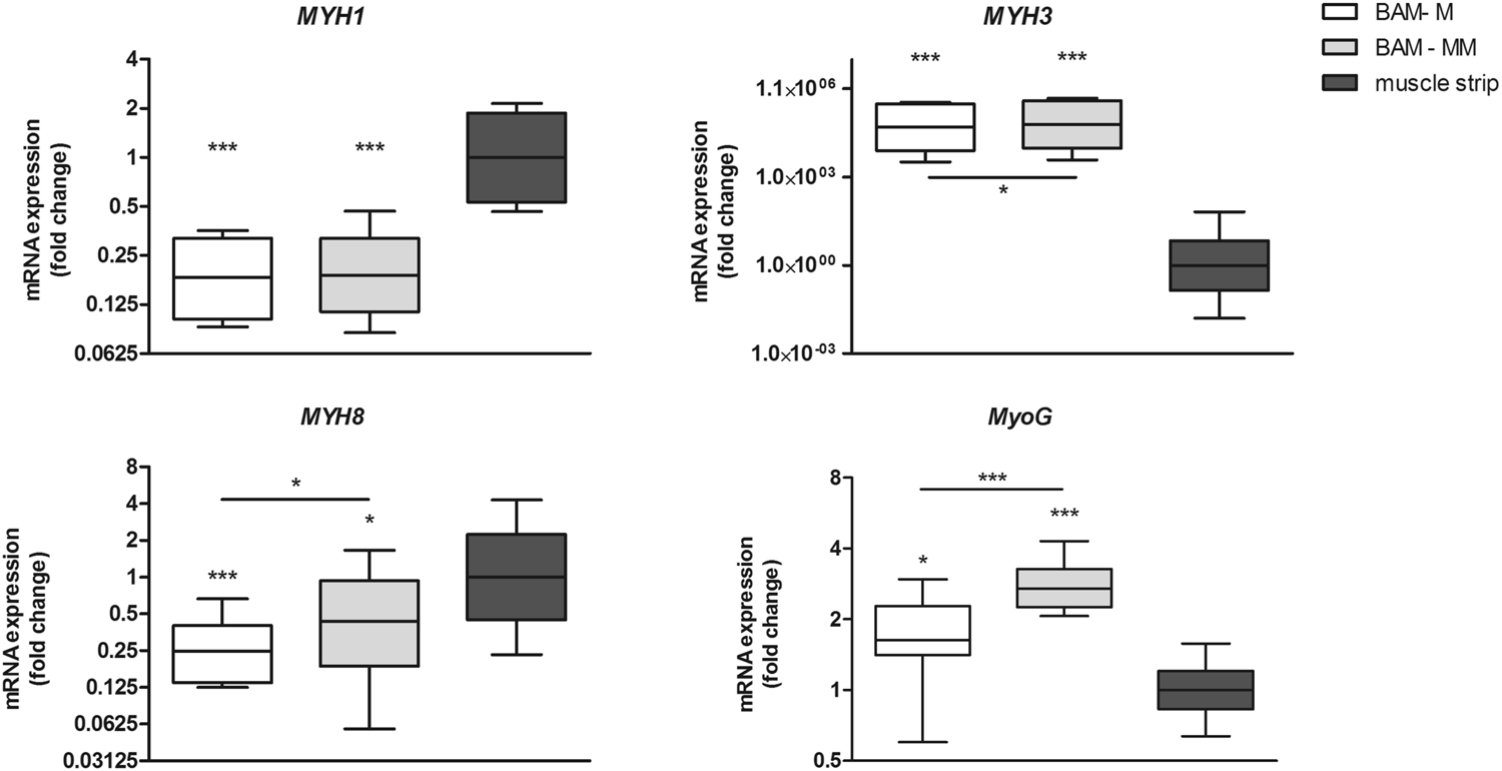
Relative myogenic gene expression in BAM-M and BAM-MM in comparison to human muscle. Relative expression of MYH isoforms and myogenin in 1 week old BAMs with myoblasts only (BAM-M) and in BAMs with a mixed muscle cell population (BAM-MM). Expression levels of these genes were quantified by quantitative real-time PCR as the fold changes relative to gene expression in muscle strips. *GAPDH, HSP90AB1 and RPL13A* were used as reference genes to normalize for the amount of cells. Relative expression in BAM-M was compared to relative expression in BAM-MM as well. Relative expression is depicted as Whisker boxplots. *p < 0.05, **p < 0.01, ***p < 0.001.

Quantitative real-time PCR was used to analyze relative expression levels of *MYH1*, *MYH3*, *MYH8* and myogenin in human BAMs in comparison to human muscle. In comparison to mature muscle (n = 5), BAM-M (n = 4) and BAM-MM (n = 5) expressed relatively lower levels of *MYH1* (5 times lower for both BAM-M and BAM-MM) and *MYH8* (4 and 2.2 times lower for respectively BAM-M and BAM-MM), showing a rather high expression of the mature *MYH1* and perinatal *MYH8* isoforms. Furthermore, myofibers within tissue engineered BAMs have significantly higher levels of embryonic *MYH3* and myogenin than in mature muscle (Fig. 4). When comparing BAM-M to BAM-MM myofibers (Fig. 4), we observe that BAM-MM shows a slightly higher expression of *MYH8, MYH3* and myogenin, suggestive for a more active myofiber formation in the BAM-MM.

### The 3D BAM model adequately reflects compound toxicity by creatine kinase release

**Figure 5:**
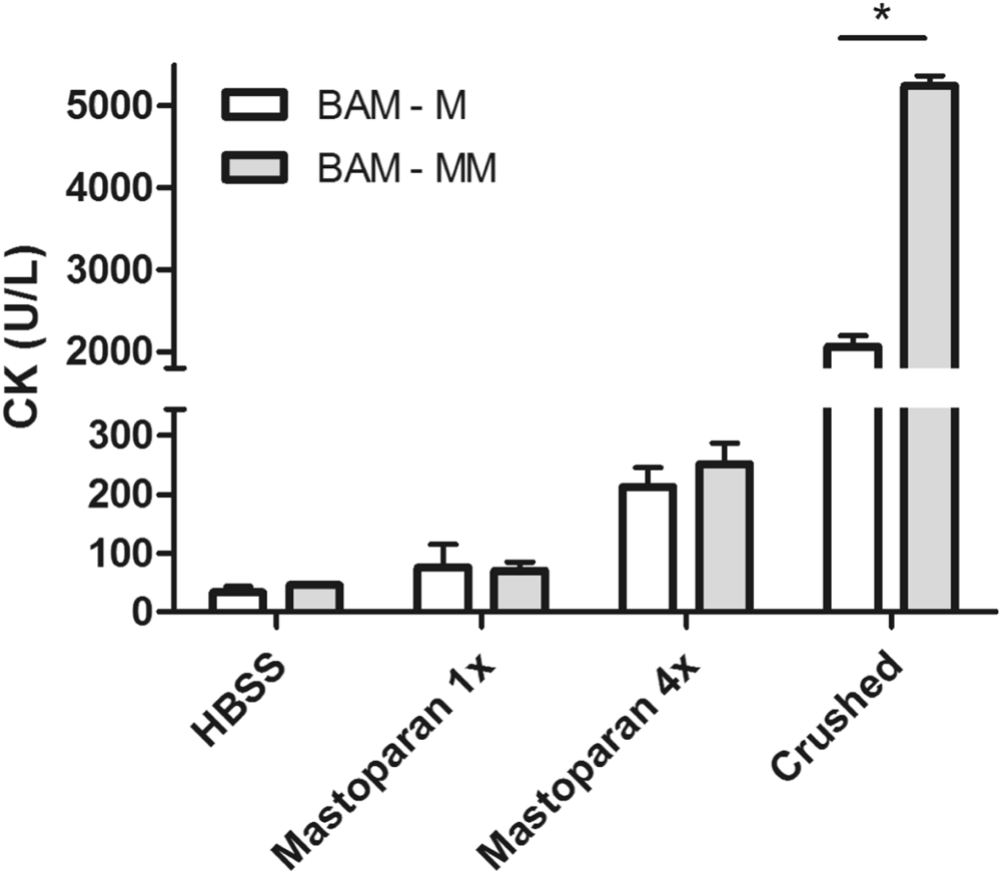
Creatine kinase release from the BAM-M and BAM-MM reflects increasing toxicity caused by increased damage from injected compounds to the BAM. Micro-injection of HBSS (1 × 1 µl) and mastoparan (1 mg/ml, 1 × 1 µl and 4 × 1 µl) as well as crushing the BAM were used to inflict an increasing amount of damage to the BAM. The creatine kinase (CK) released from the BAM during 5 minutes is a measure for toxicity/damage. Error bars depict standard deviations.

Creatine kinase is an intracellular enzyme abundant in skeletal muscle and is released upon cell damage. In the 3D injection model creatine kinase (CK) will be released into the medium due to cellular damage, when an injected compound is toxic. As a toxic compound, we used mastoparan, a wasp venom peptide with proven myotoxic effects^42^. In the proposed 3D BAM model system, toxicity was increased as follows. Injection of HBSS (4 times 1 µl) was used to control for the possibly damaging effect of the injection procedure. Second, 1 µl mastoparan (1 mg/ml) was injected once or four times in a BAM to increase the toxic effect. Finally BAMs were crushed with a cell homogenizer in order to release a maximal amount of CK. A dose-dependent increase of CK release was observed both in BAM-M and in BAM-MM when inducing increasing amounts of damage to the BAMs (Fig. 5, n = 3 for BAM-M and n = 4 for BAM-MM for each condition). Similar results were obtained in a pilot experiment in which mouse BAMs were injected (Supplementary Figure S2). A significant higher CK content was observed in BAM-MM in comparison to BAMs with myoblasts only upon crushing. CK levels measured in 800 µl HBSS incubated for 5 minutes with non-injected BAMs were below detection limit (<7 U/L, data not shown).

### Injected CDFDA is released over a time course of 40 hours in BAM

Many drugs are metabolized in the human body and a reaction that often occurs is (ester) hydrolysis. As a model compound for this reaction, we used the fluorogenic probe substrate CDFDA. This compound is hydrolyzed by intracellular esterases to the fluorescent CDF. Any unchanged CDFDA that is released by the BAM can also be measured by increasing the pH of the sample, which induces forced hydrolysis.

Before performing our experiments, we optimized the assay protocol. First, we confirmed that CDF and CDFDA were completely soluble in 30% PEG-400 in HBSS at the concentrations used for injection (500 µM). Second, we determined the detection limit and concentration range of CDF in which a linear increase of the fluorescence was recorded (0.5–100 nM, Supplementary Figure S3A). Third, the time to obtain complete forced hydrolysis of CDFDA to CDF was determined (Supplementary Figure S3B). Finally, we evaluated the occurrence of spontaneous hydrolysis of CDFDA during the experimental conditions (Supplementary Figure S3C). Since performing a freeze-thaw cycle of CDFDA samples resulted in complete hydrolysis due to a temporary increase in pH, all samples were stored in the dark in acid conditions at 4 °C until analysis.

**Figure 6:**
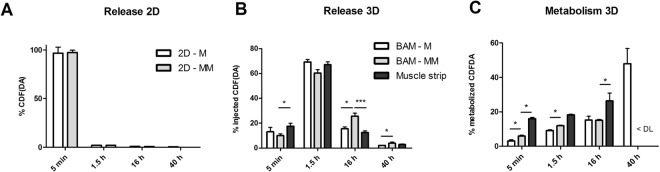
CDFDA injected in human BAMs is released in the incubation buffer (HBSS) over a time course of 40 hours. (**A**) In 2D cell culture 625 nM CDFDA solution (800 µl) was added to the differentiated myoblast only (2D-M) or mixed muscle cell population (2D-MM). Thereafter, CDFDA solution was replaced by 800 µl HBSS at 5 minutes, 1.5 h, 16 h and 40 h and CDF(DA) concentration was measured. (**B**,**C**) 500 µM CDFDA (1 µl) was injected in BAMs with myoblasts only (M), BAMs with mixed muscle cell population (MM) and human muscle strips. The release of CDF and CDFDA from BAM-M, BAM-MM and human muscle strips is shown per time point as a percentage of the total amount of effectively injected compound. (**C**) Percentage of CDFDA metabolized to CDF by the BAM. < DL indicates below detection limit. Error bars depict standard deviations. *p < 0.05, **p < 0.01, ***p < 0.001.

CDF fluorescence of samples taken at different time points (5 min, 1.5 h, 16 h, 40 h) after injection of CDFDA was measured before and after forced hydrolysis. Retention and release in BAM-M (n = 3) and BAM-MM (n = 8) were analyzed in comparison to human muscle strips (n = 7). In Fig. 6B, the release of CDF and CDFDA per time point is shown as a percentage of the total amount of effectively injected compound. It can be seen that the injected CDFDA is released into the medium over a time course of 40 hours in all conditions (Fig. 6B). Furthermore, incubation of CDFDA in 2D cell cultures does not give a release profile over time in contrast to injection in 3D BAMs (Fig. 6A). Figure 6C shows for each time point which percentage of CDFDA was hydrolyzed to CDF by the BAM-M (n = 3), BAM-MM (n = 8) and muscle strip (n = 7) at that time point. The tissue-engineered constructs are able to metabolize the injected CDFDA. The muscle strips have in general a higher metabolic capacity at each time point (Fig. 6C). At 40 hours the amount of metabolized CDFDA was below detection limit (<DL in Fig. 6C) for BAM-MM and muscle strips. CDF and CDFDA were assessed in a cell lysate after sonicating the BAMs after 40 h, but were not detectable.

### Injected pro-NanoLuc^®^ substrate is released over a time course of 40 hours

**Figure 7:**
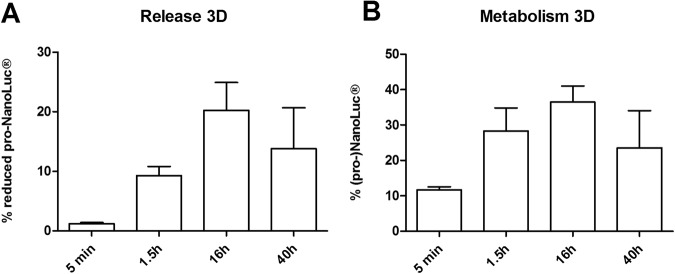
Injected pro-NanoLuc® substrate is released over a time course of 40 hours while the BAM is able to metabolize the substrate to a reduced form. 4 × 0.5 µl pro-NanoLuc® substrate was injected in 3 BAMs with myoblasts only. In (**A**), the measured release of pro-NanoLuc® substrate and NanoLuc® substrate is shown per time point as a percentage of the total amount of effectively injected compound while in (**B**) the percentage of the substrate reduced by the BAM is shown. Error bars depict standard deviations.

Another reaction in the body aimed to neutralize toxins or process drugs is reduction. As a model compound for this reaction we injected the pro-NanoLuc® substrate. This compound is a substrate of the NanoLuc® luciferase, but only after it gets reduced. During the resulting enzymatic reaction, light is produced that can be detected by a luminescence plate reader. Any pro-NanoLuc® substrate that is released without reduction by the BAM in the medium can also be measured by adding a strong reducing agent such as DTT. Samples are again taken at different time points after injection (5 min, 1.5 h, 16 h, 40 h) in BAMs with myoblast only (n = 3). Immediately before the measurement, the NanoLuc® luciferase is added to the samples and the luminescence is recorded. In the samples where DTT is added, both pro-NanoLuc® substrate and NanoLuc® substrate in the sample are measured. In Fig. 7A, the release of the (pro-)NanoLuc® substrate per time point is shown as a percentage of the total amount of effectively injected compound. The latter was measured to be 61.4 ± 7.2% of the injected amount of pro-NanoLuc® substrate. In Fig. 7B the percentage of pro-NanoLuc® substrate that was reduced by the BAM to NanoLuc® substrate is shown per time point. (Pro-)NanoLuc® substrate was assessed in a cell lysate of sonicated BAMs after 40 h, but was not detectable.

## Discussion

Increasingly 3D organoids, tissue engineered constructs and related model systems are developed for drug discovery research. A search on Pubmed with the combined subjects ‘tissue engineering’ and ‘drug’, shows 150 papers published in the year 2000 versus 2468 publications in 2016. We investigated the use of a human 3D muscle model for intramuscular injections of drugs. Drug metabolism, release and toxicity are complex processes, which are up until today mainly studied in animal models. Although animals are still needed for a full understanding of the intramuscular injection of a drug and its effects on the whole-body physiology, some important aspects may be modeled in tissue engineered constructs.

After intramuscular injection of a compound it will often be metabolized by oxidation, reduction and/or hydrolysis, mainly in the liver, but also in muscle or other tissues. About 5–7% of drugs are administered as a prodrug and need to be metabolized in order to obtain the active compound^47^. We selected two model compounds that are metabolized in two different ways in the muscle, by intracellular esterase or reductase enzymes. These were CDFDA and pro-NanoLuc® substrate, of which the hydrolyzed or reduced form can easily be measured. Although CDF is easily retained in the cell due to its negative charges, it is subject to transporter-mediated efflux typically by transporters of the multidrug resistance-associated protein (MRP) family^48,49^. This qualifies CDF as a suitable probe for our study. Depending on the nature of the injected drugs and desired sensitivity, other bioanalytical methods such as liquid chromatography coupled to tandem mass spectrometry (LC-MS/MS) or other forms of spectroscopy can be envisioned.

In the process of optimizing the micro-injection of BAMs, several parameters were evaluated. First, the thickness of the BAMs was varied. We hypothesized that thicker BAMs may allow for injection of larger volumes. Therefore we investigated increased concentrations of extracellular matrix proteins (in this case, fibrinogen) and increased numbers of embedded muscle cells. However, if the BAMs became too thick, differentiation capacity decreased, muscle fibers were less well aligned and the presence of muscle fibers in the center of the BAM decreased (Supplemental Figure S1). Based on these data, we continued working with BAMs made with 1 mg/ml fibrin, having a thickness of just below 2 mm at the time of injection. In these BAMs, we were able to inject volumes up to 1 µl per injection site. Second, we optimized the concentration of injected compound and its metabolite in order to be detectable by readily available methods. A third factor was minimizing the impact on the release profile of direct reflux of the compound in the surrounding medium while removing the needle. This immediately refluxed compound could clearly be visualized when injecting a colored compound such as Trypan blue (Fig. 1c). Therefore, we performed two washing steps after injection (resp. 1 and 2.5 min after injection) to remove refluxed compound. Since the injection volume is limited to 1 µl, injecting up to 5 times in a single BAM allowed to increase the total amount of compound injected in the BAM. Fourth, tissue exerts an elastic resistance to injection. By decreasing the outer diameter of the capillary needle from 100 µm to 10–30 µm, less resistance and better penetration into the muscle tissue was obtained. Finally, spontaneous hydrolysis of CDFDA which occurs more rapidly at alkaline pH^48^, was avoided by keeping samples at 4 °C in acidic conditions (Figure S3). A freeze-thaw cycle was also avoided because a temporary increase in pH can occur during thawing of a sample. Spontaneous reduction of the pro-NanoLuc® substrate was not observed.

After optimizing the injection procedure, we evaluated 2 different cell populations as source for myofiber formation in BAMs: a monoculture of human myoblasts (M) and an isolated human mixed muscle cell population (MM), containing 82 ± 5% desmin positive myoblasts. The remaining 18% are non-myogenic cells, mainly fibroblasts^45,46^. The presence of fibroblasts is important during myogenesis as shown in a fibroblast ablation study in mice *in vivo*^50^. For *in vitro* muscle formation, non-myogenic cells play a critical role in myogenic cell differentiation, force generation and matrix remodeling^45,51^. In 2D, myofiber formation was better in the mixed muscle cell population compared to myoblasts only. A fusion index of 64% and 80% was observed in respectively M and MM cells. Additionally when comparing M and MM cells for the number of nuclei per myofiber, we could observe 8 and 10 nuclei respectively. To better characterize the myofiber formation in BAMs, myosin heavy chain (MHC) and tropomyosin (TM) immunofluorescence stainings were performed on whole tissue. Both proteins play important roles in muscle force generation^52^. Myofibers present in both BAM-M and BAM-MM are MHC and TM-positive showing presence of occasionally striated myofibers, although to a lesser extent than in mature muscle (Fig. 3b and d). Another characteristic of skeletal muscle maturity is the presence of nuclei at the periphery of the myofibers. In our tissue-engineered human BAMs nuclei have a central location in the myofibers (Fig. 3a,c,g,h), an indication of the early myotube stage^53^.

Furthermore quantitative real-time PCR was used to study myogenic gene expression in the 2 types of BAMs in comparison to gene levels in mature human muscle. Relative expression levels of *MYH1*, *MYH3*, *MYH8* and myogenin were analyzed. Myosin heavy chain isoforms reflect the developmental state of muscle fibers^36^. *MYH3*, *MYH8* and *MYH1* are expressed in embryonic, perinatal and adult stages, respectively. Analysis of isoforms and expression levels estimates the level of maturation of myofibers present in BAMs. Myogenin expression is indicative for early myogenic differentiation and myofiber formation. Analysis showed that myofibers present in the 2 types of BAM express 5 times lower levels of *MYH1* compared to myofibers in adult muscle and 4 and 2.2 times lower levels of *MYH8* for respectively BAM-M and BAM-MM, indicating that myofibers are not as mature as in adult muscle. High expression of *MYH3* and myogenin in BAMs shows early myotube formation resulting in more embryonic myofibers. Comparing BAM-MM to BAM-M, the significantly higher *MYH8*, *MYH3* and myogenin levels indicate a more active myofiber formation in BAM-MM. Also significant higher CK content was observed in BAM-MM. Creatin kinase expression is highly increased during myogenesis and reflects the maturity grade of myofibers^54,55^. From the above, we can conclude that both BAM-M and BAM-MM have clear myogenic features but are less mature than adult muscle and that BAM-MM shows more pronounced differentiation than BAM-M.

The release kinetics of the two injected compounds was clearly different. While already ∼80% of effectively injected CDFDA was released after 1.5 hours for BAM-M, this was only ∼40% in the case of the pro-NanoLuc® substrate (Figs 6 and 7). This could be due to the fact that the pro-NanoLuc® substrate was a suspension instead of a solution as was the case with CDFDA. In a suspension, undissolved particles are present, and gradual dissolution typically causes a slower release of the compound. A suspension for injection represents an interesting dosage form for certain drugs. For example, a slow-release version of antipsychotic drugs such as paliperidone may decrease the number of individual injections needed, leading to a better therapy compliance^56^. Furthermore we could clearly show the utility of our 3D model to follow release kinetics over time, in constrast to a 2D cell culture model (Fig. 6A and B), emphasizing the benefits of the BAM model. The release of CDF(DA) in BAMs and human muscle strip was highest at 1.5 hours and had a similar release profile over time. Overall, release kinetics from the muscle can indeed be an important parameter in drug development, which can be simulated in this *in vitro* model.

The metabolic turnover was similar between the two compounds, up to 16 hours after injection. The longer the compounds were present in the BAM, the higher the ratio of metabolite over parent compound. The muscle strips with mature myofibers had in general a higher metabolic capacity per time point. It is well-known that intracellular aspecific esterases can hydrolyze compounds such as CDFDA^57^ while reductases play a role in the reduction of the pro-NanoLuc® substrate, as shown for prodrugs that are activated by reduction^58–60^. Part of the hydrolyzed CDFDA was due to spontaneous hydrolysis as shown in Figure S3C.

The next step in drug development is translating the *in vitro* results to corresponding *in vivo* exposures in the body. This can be achieved by *in vitro in vivo* extrapolation (IVIVE) linked to physiologically based pharmacokinetic (PBPK) modeling^61^. IVIVE-PBPK allows to predict the *in vivo* exposure profiles in the target tissue, relying on all relevant processes related to the absorption, distribution, metabolism and excretion of the drug of interest. Furthermore, this so-called ‘bottom-up’ modelling and simulation approach, based on data obtained in *in vitro* tests, enables to determine adequate drug doses yielding desired target concentrations *in vivo*^62^. To apply an IVIVE approach to the BAM model also scaling factors between *in vitro* and *in vivo* muscle tissue (e.g. amount of muscle tissue *in vitro*/*in vivo*) are required, in addition to the *in vitro* data on drug release and metabolism after BAM injections. At the PBPK level, muscle physiological parameters such as muscle blood perfusion rates need to be taken into account. *In vivo* pharmacokinetic data for IM haloperidol decanoate administration are available^27^, and could be used as reference data to evaluate the value of a retrospective prediction based on IVIVE-PBPK. Such an exercise will support the validation of the BAM model as a suitable representation of the *in vivo* muscle environment.

Some challenges regarding the use of tissue-engineered constructs for drug research remain^63^. First, the need to reflect complex organ functions. Regarding skeletal muscle, this encompasses the coculture of different tissue types such as nerve tissue, connective tissue and blood vessels. Coculturing these different tissue types and obtaining functionality is currently still a challenge^33,64–66^. The BAM models described in this paper only consist of a monoculture of myoblasts or a mixed muscle cell population and are able to evaluate the effects of injected compounds on myofibers. To better reflect the whole-muscle physiology, we aim to include multiple cell types in the BAM model. We are developing a skeletal muscle model in which endothelial precursor cells are added to induce a vascular network in the BAM^33^. The presence of a vascular network and blood flow determines the local drug absorption and therefore the corresponding drug concentration and release time in the muscle. For example, the difference in blood flow between muscle groups resulted in different plasma concentrations and release time of intramuscular injected lidocaine^67,68^. To address this challenge one could use a bio-reactor to obtain flow perfusion in the construct and mimic blood flow^64,69^. Second, *in vivo* tissues are stimulated by chemical, electrical and mechanical stimuli from their environment to differentiate/mature into physiologically relevant tissues. Our *in vitro* tissue-engineered skeletal muscle could also benefit from mechanical and electrical stimulation resulting in more mature striated skeletal muscle tissue^34,70,71^. Third, an inherent characteristic of this model is the absence of other organs, such as the liver or the immune system. Influence of immune cells on pharmacokinetics and –dynamics may be important e.g. for vaccine testing. It has been shown that macrophages are capable of *in vivo* (pro)drug biotransformation^72^. Approaches to integrate such responses are under development, typically on a microscale for screening purposes and are referred to as organ-on-a-chip systems. These are micro-engineered 3D models of the functional units of human organs that are connected with microfluidic channels and mimic organ-organ interactions^5^. An important application of these model systems is testing a more realistic and overall effect of new drugs on several organs of the human body simultaneously^73^. Although animal experiments can still be justified to monitor complex interactions, tissue model systems can partially reduce this need. Finally, since producing tissue-engineered constructs is labor intensive and time consuming, there is a need for automated procedures. An automated drug screening setup, as described previously to assess force of engineered muscle^37^, with an additional micro-injection apparatus and sampling, would allow for higher throughput and decreased variability.

To conclude we can state that, using our *in vitro* 3D tissue engineered model for intramuscular injection, we are able to evaluate different parameters such as compound toxicity, release and biotransformation. The proposed system addresses the need to minimize animal experimentation.

## Electronic supplementary material

Supplementary information
