# Supplementary material for: Human tissue-engineered skeletal muscle: a novel 3D in vitro model for drug disposition and toxicity after intramuscular injection
Source: Sci Rep. 2018 Aug 15;8:12206. doi: 10.1038/s41598-018-30123-3 (PMC6093918; doi:10.1038/s41598-018-30123-3)
Supplement: Supplementary file 1 — Supplementary information [file 41598_2018_30123_MOESM1_ESM.pdf]

SUPPLEMENTARY INFORMATION

Human tissue-engineered skeletal muscle: a novel 3D *in vitro* model for drug disposition and toxicity after intramuscular injection

D Gholobova[a,$], M Gerard[a,$], L. Decroix[a,b], L Desender[a], N Callewaert[c], P Annaert[d], L Thorrez[a,*]

[a] Tissue Engineering Lab, Department of Development and Regeneration, KU Leuven, E. Sabbelaan 53, 8500 Kortrijk, Belgium

[b] Current affiliation: Faculty of Physical Education and Physiotherapy, Department of Human Physiology and Sportsmedicine, Building L, Pleinlaan 2, Brussels, Belgium

[c] AZ Groeninge, President Kennedylaan 4, 8500 Kortrijk, Belgium

[d] Drug Delivery and Disposition, Department of Pharmaceutical and Pharmacological Sciences, KU Leuven, O&N II Herestraat 49 - box 921, 3000 Leuven, Belgium

[$]Shared first authors

* Corresponding author: Prof. Lieven Thorrez, email: lieven.thorrez@kuleuven.be, tel: +32 56 24 62 31, Fax: +32 56 24 69 94

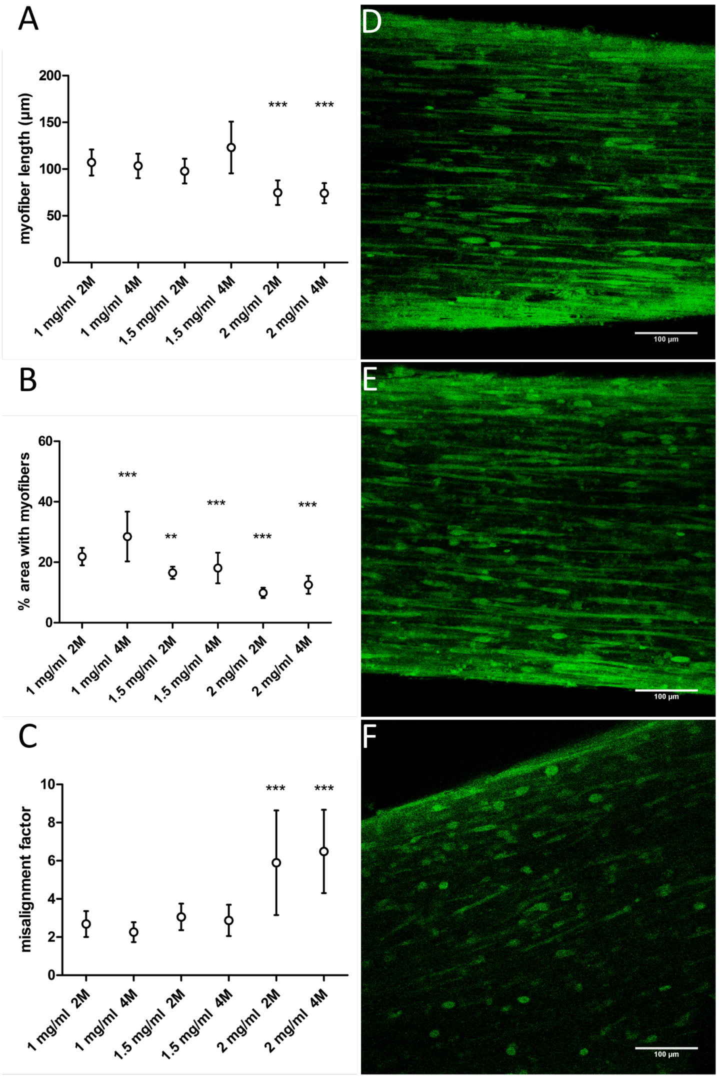

Figure S1. Myofiber formation in C2C12 BAMs with different fibrin concentration and cell numbers. (A-C) Parameters characterizing the formation of myofibers in C2C12 fibrin BAMs with different fibrin concentrations (1 mg/ml, 1.5 mg/ml or 2 mg/ml) and total cell numbers (2.10^6 or 4.10^6). Multiple comparisons were made by a Kruskal Wallis test followed by a Dunn's post test against the condition with 1 mg/ml fibrin and 2.10^6 total cell number condition. All values were expressed as mean ± standard deviation. * $p< 0.05$, ** $p< 0.01$, *** $p< 0.001$ (D-F). Fluorescent confocal 2D images of GFP labeled myofibers in C2C12 BAMs containing a constant total cell number (4.10^6) and an increasing fibrin concentration (1 mg/ml (D), 1.5 mg/ml (E) and 2 mg/ml (F)). Scale bars represent 100 µm.

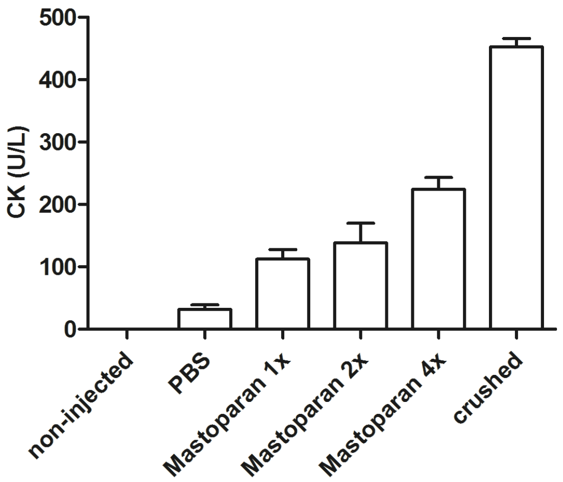

Figure S2. Creatine kinase release from the C2C12 BAM reflects increasing toxicity caused by increased damage from injected compounds to the BAM. Micro-injection of PBS (1x 0.8 µl, n = 4) or mastoparan (1mg/ml, 1x (n = 5), 2x (n = 3) or 4x (n = 2) 0.8 µl) as well as crushing the BAM (n = 6) were used to inflict an increasing amount of damage to the mouse BAM. After injection(s), the mouse BAMs were submerged in 800 µl PBS. After 5 minutes, the HBSS was removed and stored at -21°C. The creatine kinase released from the BAM during these 5 minutes or after crushing, is a measure for toxicity/damage. Creatine levels measured in 800 µl HBSS incubated for 5 minutes with non-injected mouse BAMs (n = 6) is shown as a negative control. Error bars depict standard deviations.

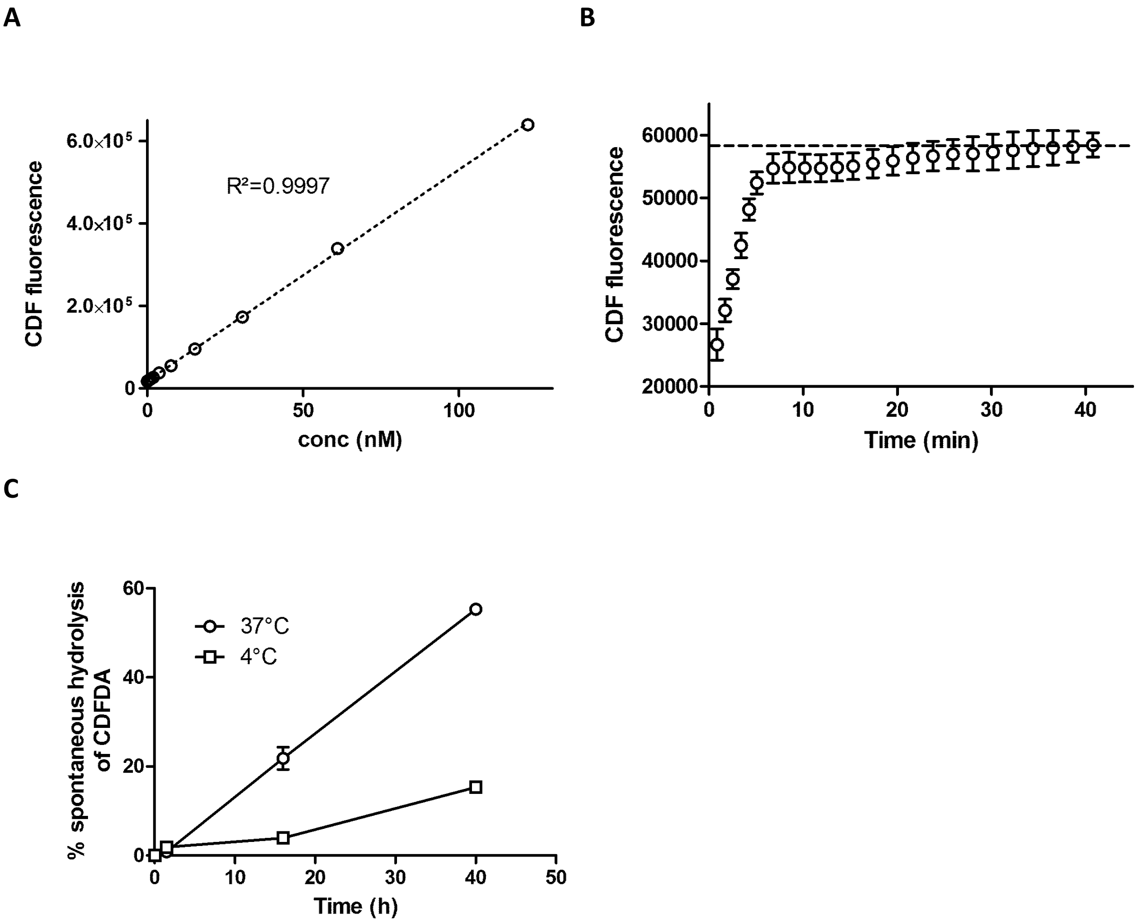

Figure S3. Experimental conditions of CDF detection and CDFDA hydrolysis. (A) Calibration curve of CDF fluorescence versus its concentration in nM. Every data point is an average of 3 measurements (SD shown but invisible due to small variation). A linear relationship between the two parameters can be seen between 0.5 and 100 nM. In (B), the time to obtain complete hydrolysis of CDFDA to CDF was measured at room temperature. CDFDA was diluted to a concentration of 200 nM and 6 wells were filled with 150 µl of this solution. To each well, 3 µl NaOH (2M) was added to obtain a pH ≥ 11 and the plate was shaken for 30 sec. Thereafter, the plate was measured each 25 seconds for 100 times. After 30 minutes, the plateau of CDF fluorescence is reached (dotted line). For clarity reasons, not all data points are shown on the graph. (C) Spontaneous hydrolysis of CDFDA (100 nM) during incubation (37°C, 5% CO_2) and storage in HBSS at 4°C during 40 hours.

Table S1

	[CDF] measured	% recovery
**A. 625 nM non-hydrolyzed CDFDA**	10.5 ± 1.9	1.7 ± 0.3
	10.3± 2.2	1.6 ± 0.4
**B. 625 nM non-hydrolyzed CDFDA, washed with 0,5% Triton-X 100**	< det lim	< det lim
	< det lim	< det lim
**C. 625 nM CDFDA hydrolyzed to CDF**	344.6 ± 3.0	55.1± 0.5
	347.0 ± 21.5	55.5± 3.4
**D. 625 nM CDFDA hydrolyzed to CDF, washed with 0,5% Triton-X 100**	542.5± 33.7	86.8± 5.4
	515.9± 20.2	82.5± 3.2

Table S1. Characterization of CDFDA adherence to silicone mold. A CDFDA solution of 625 nM (800 µl) was incubated in a silicone mold in a 6-well plate for 6 hours at room temperature. Condition A: the concentration of CDF in the CDFDA solution was measured after 6 hours. This is the spontaneously hydrolyzed CDFDA. Condition B: Identical to condition A, but the mold was washed with 800 µl HBSS with 0.5% Triton-X-100 to remove any CDFDA that adhered to the silicone mold. Condition C: the CDFDA recovered from the mold was hydrolyzed to CDF using a pH jump to 11. This is the total concentration of CDFDA and CDF that was present in the sample. Condition D: Identical to condition C, but the mold was washed with 800 µl HBSS with 0.5% Triton-X-100. All numbers are the average of three technical replicates with their standard deviation. Two replicate samples were measured and are shown for each condition.
